# Exploring biomarkers for diagnosing and predicting organ dysfunction in patients with perioperative sepsis: a preliminary investigation

**DOI:** 10.1186/s13741-024-00438-z

**Published:** 2024-07-24

**Authors:** Linghui Jiang, Shiyu Chen, Shichao Li, Jiaxing Wang, Wannan Chen, Yuncen Shi, Wanxia Xiong, Changhong Miao

**Affiliations:** Department of Anaesthesiology, Zhongshan Hospital, Fudan University, Shanghai, 200032 China

**Keywords:** Sepsis, Organ dysfunction, Perioperative period, Diagnostic marker, Predictive marker

## Abstract

**Objective:**

Early diagnosis and prediction of organ dysfunction are critical for intervening and improving the outcomes of septic patients. The study aimed to find novel diagnostic and predictive biomarkers of organ dysfunction for perioperative septic patients.

**Method:**

This is a prospective, controlled, preliminary, and single-center study of emergency surgery patients. Mass spectrometry, Gene Ontology (GO) functional analysis, and the protein-protein interaction (PPI) network were performed to identify the differentially expressed proteins (DEPs) from sepsis patients, which were selected for further verification via enzyme-linked immunosorbent assay (ELISA). Logistic regression analysis was used to estimate the relative correlation of selected serum protein levels and clinical outcomes of septic patients. Calibration curves were plotted to assess the calibration of the models.

**Results:**

Five randomized serum samples per group were analyzed via mass spectrometry, and 146 DEPs were identified. GO functional analysis and the PPI network were performed to evaluate the molecular mechanisms of the DEPs. Six DEPs were selected for further verification via ELISA. Cathepsin B (CatB), vascular cell adhesion protein 1 (VCAM-1), neutrophil gelatinase-associated lipocalin (NGAL), protein S100-A9, prosaposin, and thrombospondin-1 levels were significantly increased in the patients with sepsis compared with those of the controls (*p* < 0.001). Logistic regression analysis showed that CatB, S100-A9, VCAM-1, prosaposin, and NGAL could be used for preoperative diagnosis and postoperative prediction of organ dysfunction. CatB and S100-A9 were possible predictive factors for preoperative diagnosis of renal failure in septic patients. Internal validation was assessed using the bootstrapping validation. The preoperative diagnosis of renal failure model displayed good discrimination with a C-index of 0.898 (95% confidence interval 0.843–0.954) and good calibration.

**Conclusion:**

Serum CatB, S100-A9, VCAM-1, prosaposin, and NGAL may be novel markers for preoperative diagnosis and postoperative prediction of organ dysfunction. Specifically, S100-A9 and CatB were indicators of preoperative renal dysfunction in septic patients. Combining these two biomarkers may improve the accuracy of predicting preoperative septic renal dysfunction.

**Trial registration:**

The study was registered at the Chinese Clinical Trials Registry (ChiCTR2200060418) on June 1, 2022.

**Supplementary Information:**

The online version contains supplementary material available at 10.1186/s13741-024-00438-z.

## Introduction

Sepsis is a life-threatening state of organ dysfunction caused by an abnormal response to infection and is a common cause of morbidity and mortality in perioperative patients undergoing emergency surgery (Fleischmann et al. 2016; Philippon and Freund 2019). The mortality rates of sepsis and septic shock are estimated to exceed 40% (Chiu and Legrand 2021). Alleviating the global epidemiological burden of sepsis remains difficult.

Sepsis development involves different biochemical and immunological pathways as well as altered serum protein levels (Hotchkiss et al. 2013). The sequential organ failure score (SOFA) was cited in the diagnostic procedures of Sepsis 3.0 in 2016 (Singer et al. 2016). However, researchers assessed the SOFA and concluded that the SOFA and quick SOFA were not strict sepsis screening tools because both tools reduced the sensitivity of early diagnosis and delayed early treatment for patients (González Del Castillo et al. 2017; Konradsen and Lien 2017; Simpson 2016). Therefore, new biomarkers with higher sensitivity and specificity as indicators are urgently needed (Opal and Wittebole 2020). Pierrakos and Vincent summarized that at least 178 sepsis biomarkers have been reported, of which, only 16 factors were evaluated specifically for early diagnosis of sepsis (Pierrakos and Vincent 2010). Jiao et al. found five proteins that were tightly correlated with sepsis presence, and four proteins were related to sepsis prognosis in rats (Jiao et al. 2014). However, few studies have investigated the dysfunction of different organs in septic patients, especially in those undergoing emergency surgery. Therefore, we conducted this study to identify novel biomarkers for diagnosing and predicting various organ dysfunctions in perioperative septic patients undergoing emergency surgery.

## Methods

### Study and ethics

This was a prospective, controlled, single-center study of patients with sepsis (the sepsis group) and without sepsis (the control group). The study was registered at the Chinese Clinical Trials Registry (ChiCTR2200060418) on June 1, 2022, and approved by the Ethics Review Committee (Ethics No. B2022-241(2)). All participants provided signed informed consent. The procedures followed were in accordance with the of the responsible committee on human experimentation and with the ethical guidelines of the 2003 Helsinki Declaration.

### Design, setting, and participants

From July 2022 to October 2022, septic patients were enrolled in the sepsis group. Inclusion criteria were (1) volunteered to participate in the trial and signed informed consent; (2) planned to undergo emergency surgery; (3) aged ≥ 18 and ≤ 80 years; (4) had American Society of Anesthesiologists (ASA) physical statuses I–IV; and (5) met the Sepsis 3.0 diagnostic criteria. Exclusion criteria were (1) comorbidity with tumor disease; (2) pre-existing cardiac insufficiency with left ventricular ejection fraction (LVEF) < 40% before the current infection; (3) pre-existing severe hepatic insufficiency (prothrombin ratio < 15%) before this infection; (4) pre-existing severe renal insufficiency (estimated glomerular filtration rate [eGFR] < 30 ml/min/1.73 m^2^) before this infection; and (5) pre-existing mental illness, refusal to participate or failure to sign the informed consent. Withdrawal criteria for the sepsis group were infection found to be caused by a tumor during the operation.

Patients undergoing elective surgery during the same period were included in the control group. Inclusion criteria were (1) volunteered to participate in the trial and signed informed consent; (2) ASA I–II; (3) aged ≥ 18 years and ≤ 80 years; and (4) planned to undergo surgery unrelated to infection or tumor removal. Exclusion criteria were (1) ASA ≥ III; (2) pre-existing cardiac insufficiency; (3) pre-existing severe hepatic insufficiency; (4) pre-existing severe renal insufficiency; and (5) pre-existing mental illness, refusal to participate, or failure to sign the informed consent. Withdrawal criteria for the control group were infection or tumors found in the lesion during the operation.

### Blood sampling and mass spectrometry

Venous blood samples were taken before surgery on the day of surgery (D0) for both groups and on postoperative day 7 (D7) in the sepsis group. Serum samples were collected and stored at − 80 °C. Five randomized serum samples per group were analyzed via mass spectrometry (timsTOF Pro, Bruker, Germany), and mass spectrometry databases were established for both groups to screen out the significantly altered proteins in the septic patients. RAW files were identified, quantified, and analyzed using Maxquant1.4.1.2 (Max Planck Institute of Biochemistry, Martinsried, Munich, Germany). The ion peak intensity reflected the relative protein abundances and the quantitative ratio of the proteins was normalized against the median ratio value of the internal standard sample.

### Gene ontology (GO) functional analysis and the protein­-protein interaction (PPI) network

We used the “cluster Profiler” package in R for the GO annotation of the differentially expressed proteins (DEPs). The GO database was used to analyze the cellular components, molecular function, and biological processes of the proteins (*p* value cutoff 0.05, *q* value cutoff 0.05). The PPI network of these DEPs was constructed using the Search Tool for the Retrieval of Interacting Genes (STRING) database (https://www.string-db.org/).

### DEP validation

ELISA was used to validate the candidate biomarkers obtained from the GO analysis using ELISA kits (Shanghai Xinle Biotechnology Co., Ltd., China) per the manufacturer’s instructions.

### Follow-up

Patients’ baseline clinical characteristics were recorded, including age, sex, height, weight, history of previous diseases, history of special drug use, and results of major laboratory tests such as routine blood counts (white blood cell count, neutrophil ratio hemoglobin count, platelet count), infection indicators (procalcitonin, C-reactive protein, plasma lactate, interleukin-1β, interleukin-2 receptor, interleukin-6, interleukin-8, interleukin-10, tumor necrosis factor-α), liver and kidney function parameters (bilirubin, albumin, creatinine), myocardial dysfunction markers (cardiac Troponin T, myoglobin, creatine kinase, N-terminal brain natriuretic peptide precursor), and serum electrolytes (serum sodium, potassium, calcium, magnesium). The follow-up time points were D1 (1 day after surgery), D7, at discharge, and 1 month after surgery. The last visit at 1 month was done via telephone; the other visits were on-site. Clinical data, including vital signs; laboratory tests; SOFA score; organ dysfunction; intensive care unit (ICU) admission, length of hospital stay, and discharge outcomes, were collected at each on-site follow-up visit. Multiple organ dysfunction score (Marshall et al. 1995) was used to assess organ dysfunction events. Oxygenation index, serum creatinine, serum bilirubin, platelet count, Glasgow coma scale and pressure-adjusted heart rate were selected as variables to reflect an important system functional status. The prognostic variables were septic shock incidence, organ dysfunction incidence, proportion of ICU admissions, length of hospital stay, hospital mortality, and 1-month mortality.

### Statistical analysis

Data analyses were performed in R, project 3.5.3, for Windows and IBM SPSS Statistics, version 22.0. Normality and homogeneity of variance were tested via the Shapiro-Wilk and Levene’s tests. Categorical variables are reported as frequencies and percentages. Continuous variables with a normal distribution are presented as the means ± standard deviation; all others are described as medians and interquartile range (IQR). Baseline characteristics between the groups were compared using Fisher’s precision probability test or Pearson’s chi-square test for categorical variables, using Student’s *t*-test for normally distributed continuous variables or Wilcoxon rank-sum test for non-normally distributed continuous variables. Spearman’s correlation test was conducted to measure the statistical relationship between preoperative blood tests and selected serum proteins. LR analysis was used to estimate the relative risk of selected serum protein levels and clinical outcomes of septic patients. The incidence was entered as a dependent variable, Y, in the LR model and was coded as 0 for absent (did not occur) or 1 for present (occurred). We applied and compared two regression methods: stepwise logistic regression (SL) and logistic least absolute shrinkage and selection operator (LASSO) regression. To obtain the logistic LASSO estimator, we used the glmnet package in R. For the stepwise selection, we used the Akaike information criterion to select the covariates. For the logistic LASSO regression, we used cross-validation to select λ. We calculated the area under the receiver operating characteristic curve (AUROC) for the data to measure the predictive performances of the fitted models. *P* < 0.05 was considered statistically significant.

Calibration curves were plotted to assess the calibration of the models. A significant test statistic implies that the models do not calibrate perfectly. To quantify the discrimination performance of the model, Harrell’s C-index was measured. The models were subjected to bootstrapping validation to calculate a relatively corrected C-index.

## Results

### Patient characteristics

Sixty-six patients (36 in the sepsis group and 30 controls) were included. Table 1 summarizes the patients’ characteristics. Twenty-nine septic patients were diagnosed with acute biliary tract infection and underwent biliary surgery. Five septic patients had intestinal infections and underwent partial intestinal resection. The remaining two septic patients underwent an abscess incision. The preoperative SOFA and APACHE II scores were significantly higher in the sepsis group than in the control group (0.45 ± 0.68 vs 7.20 ± 5.37 and 2.63 ± 2.11 vs 16.00 ± 7.80, respectively). The same trend was found for the C-reactive protein (CRP) and procalcitonin (PCT) (*p* < 0.05).

**Table 1:** Baseline characteristics of septic patients and controls (*p < 0.05)

**Variable**	**Control**	**Sepsis**
Number	30	36
Age (years)	59.62 ± 5.82	69.51 ± 9.71
Male (%)	49%	45%
Acute Physiology and Chronic Health Evaluation-II Score	2.63 ± 2.11	16.00 ± 7.80*
Sequential Organ Failure Assessment Score	0.45 ± 0.68	7.20 ± 5.37*
Site of infection		
Biliary tract	NA	29
Intestinal tract	NA	5
Others	NA	2
Patients with organ insufficiency	0	36
Length of ICU stay (day)	0	22.5 ± 15.4*
Death during ICU	0	4
WBC (× 10^9^/L)	7.31 ± 2.03	14.44 ± 5.47*
Procalcitonin (μg/L)	0.22 ± 0.05	2.86 ± 0.58*
C-reactive protein (mg/L)	1.00 ± 0.76	170.97 ± 86.24*

### DEP identification and selection

**Fig. 1:**
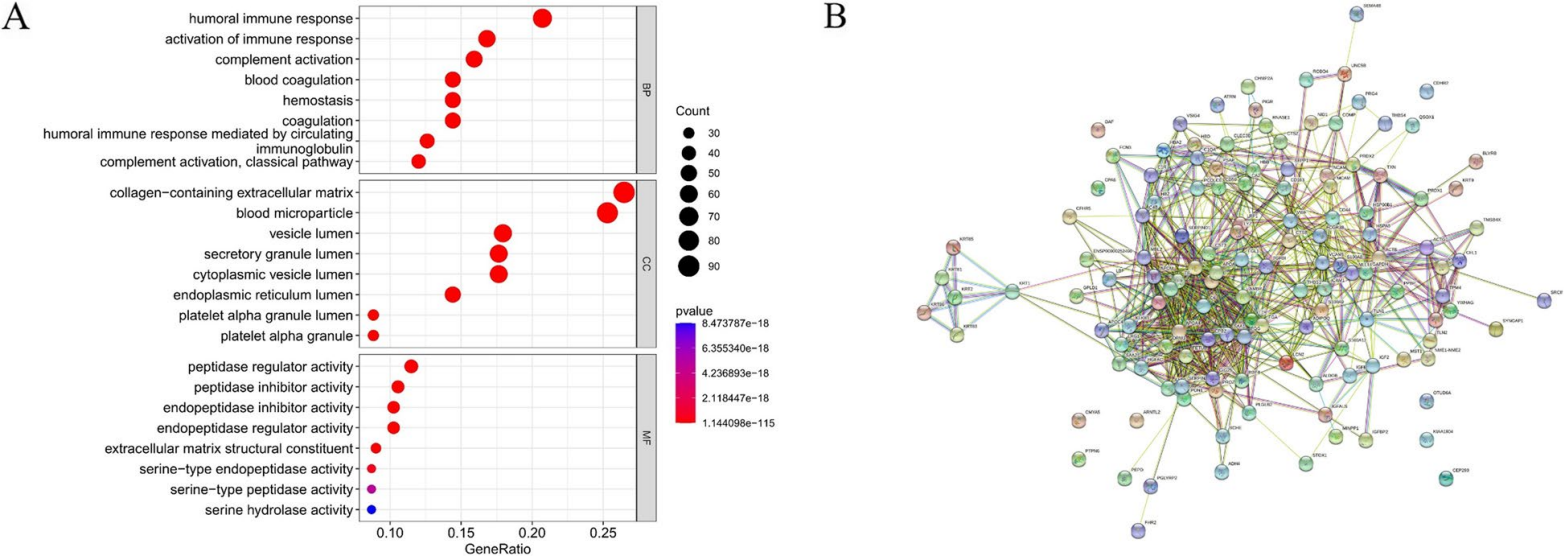
Functional enrichment analysis of the differentially expressed serum proteins in sepsis patients. **A** significantly enriched GO terms of the differentially expressed proteins; (**B**) the protein-protein interaction network constructed with the differentially expressed proteins

Compared with those of the control group, 146 proteins differed significantly (*P* < 0.05) in the sepsis group, of which, 64 were upregulated, and 82 were downregulated on mass spectrometry (Supplemental Figure S1A, B). These 146 DEPs were analyzed via GO and the PPI network (Fig. 1A, B). For biological processes, most DEPs were involved in humoral immune response, complement activation, blood coagulation, and hemostasis. Finally, we selected six DEPs that were closely associated with sepsis: cathepsin B (CatB), vascular endothelial cell adhesion molecule (VCAM-1), neutrophil gelatinase-associated lipoprotein (NGAL), protein S100-A9, prosaposin, and thrombospondin-1 (TSP-1).

### DEP validation

We further verified the expressions of the six candidate biomarkers in the septic patients and controls via ELISA. CatB, VCAM-1, NGAL, S100-A9, prosaposin, and TSP-1 levels were significantly increased in the sepsis group (*p* < 0.001) compared with those of the controls (Table 2).

**Table 2:** Preoperative levels of selected serum proteins between sepsis and control group

	Control (*n* = 30)	Sepsis (*n* = 36)	*p* value
S100A9 (ng/mL)	43.72 ± 12.69	85.85 ± 20.57	< 0.001
prosaposin (ng/mL)	1.47 ± 0.38	3.43 ± 1.29	< 0.001
VCAM-1^*a*^ (ng/mL)	223.56 ± 58.03	445.78 ± 147.37	< 0.001
thrombospondin-1 (ng/mL)	129.44 ± 25.81	249.85 ± 80.36	< 0.001
NGAL^*b*^ (ng/mL)	90.51 ± 30.14	156.80 ± 65.17	< 0.001
CatB^*c*^ (ng/mL)	1.515 ± 0.39	2.957 ± 1.05	< 0.001

^*a*^Vascular cell adhesion protein 1

^*b*^Neutrophil gelatinase-associated lipocalin

^*c*^Cathepsin B

### Correlation analysis of six preoperative biomarkers and biochemical parameters in septic patients

**Fig. 2:**
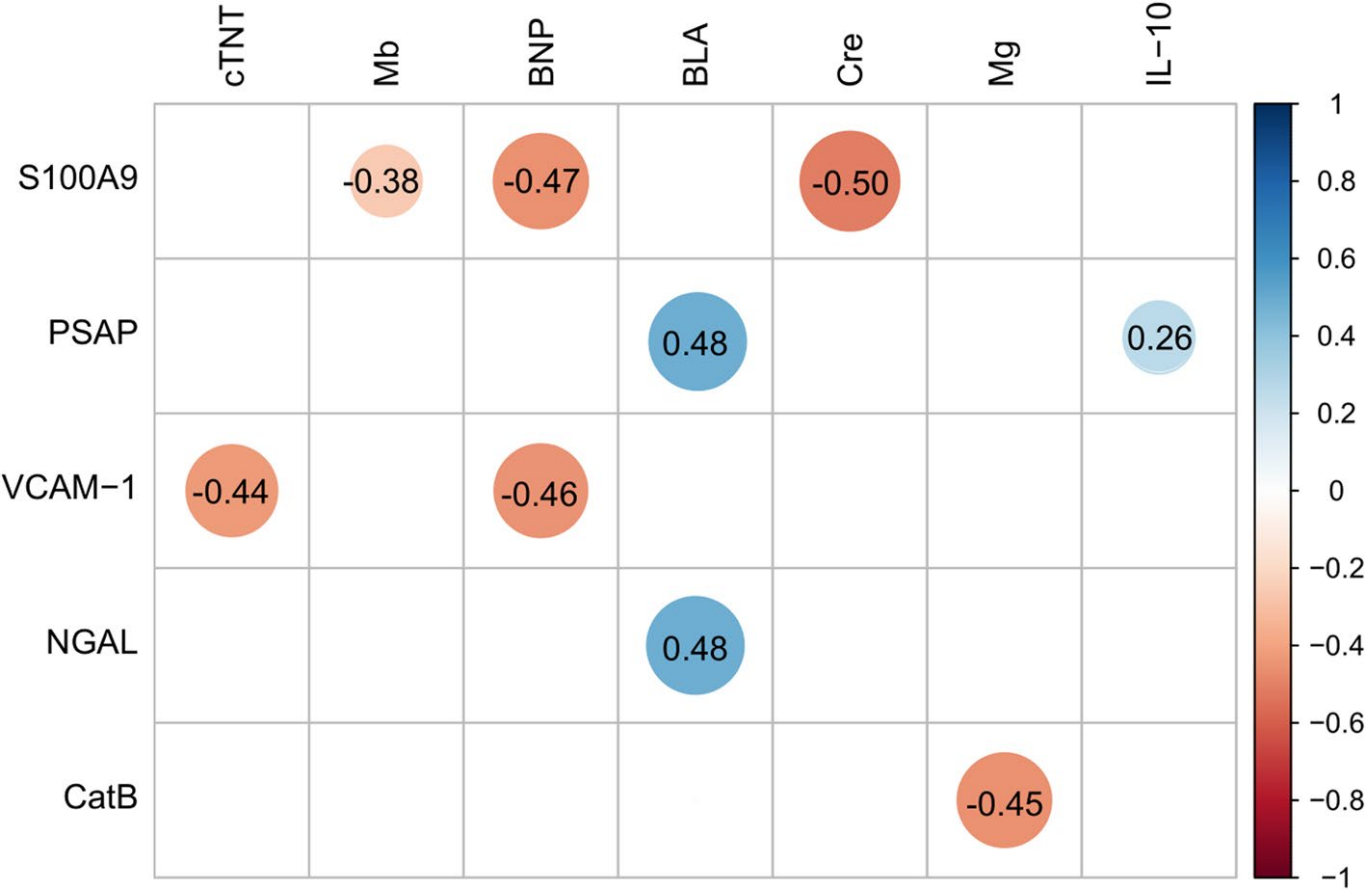
Preoperative blood tests and selected serum proteins. The correlogram shows correlation coefficients for all pairs of variables, with more intense colors for more extreme correlations. The plot below indicated those whose correlations were statistically significant

Serum levels of the above six biomarkers obtained immediately upon admission were considered the preoperative baseline in the sepsis group. Organ dysfunction events were diagnosed according to the preoperative organ function status using the multiple organ dysfunction score. Figure 2 shows the Spearman correlation analysis of the six preoperative biomarkers and other biochemical parameters with preoperative organ dysfunction in the septic patients as well as the significant parameters (*p* < 0.05). S100-A9 was strongly correlated with myoglobin (*r*_s_ =  − 0.38), brain natriuretic peptide (BNP) (*r*_s_ =  − 0.47), and creatinine (*r*_s_ =  − 0.50). Prosaposin was strongly correlated with plasma lactate (*r* = 0.48) and interleukin 10 (IL-10) (*r*_s_ = 0.26). VCAM-1 was strongly correlated with cardiac troponin T (cTNT) (*r*_s_ =  − 0.44) and BNP (*r*_s_ =  − 0.46). NGAL was strongly correlated with plasma lactate (*r*_s_ = 0.48). CatB was strongly correlated with serum magnesium (*r*_s_ =  − 0.45).

### Correlation between preoperative biomarkers and preoperative single organ dysfunction in septic patients

**Fig. 3:**
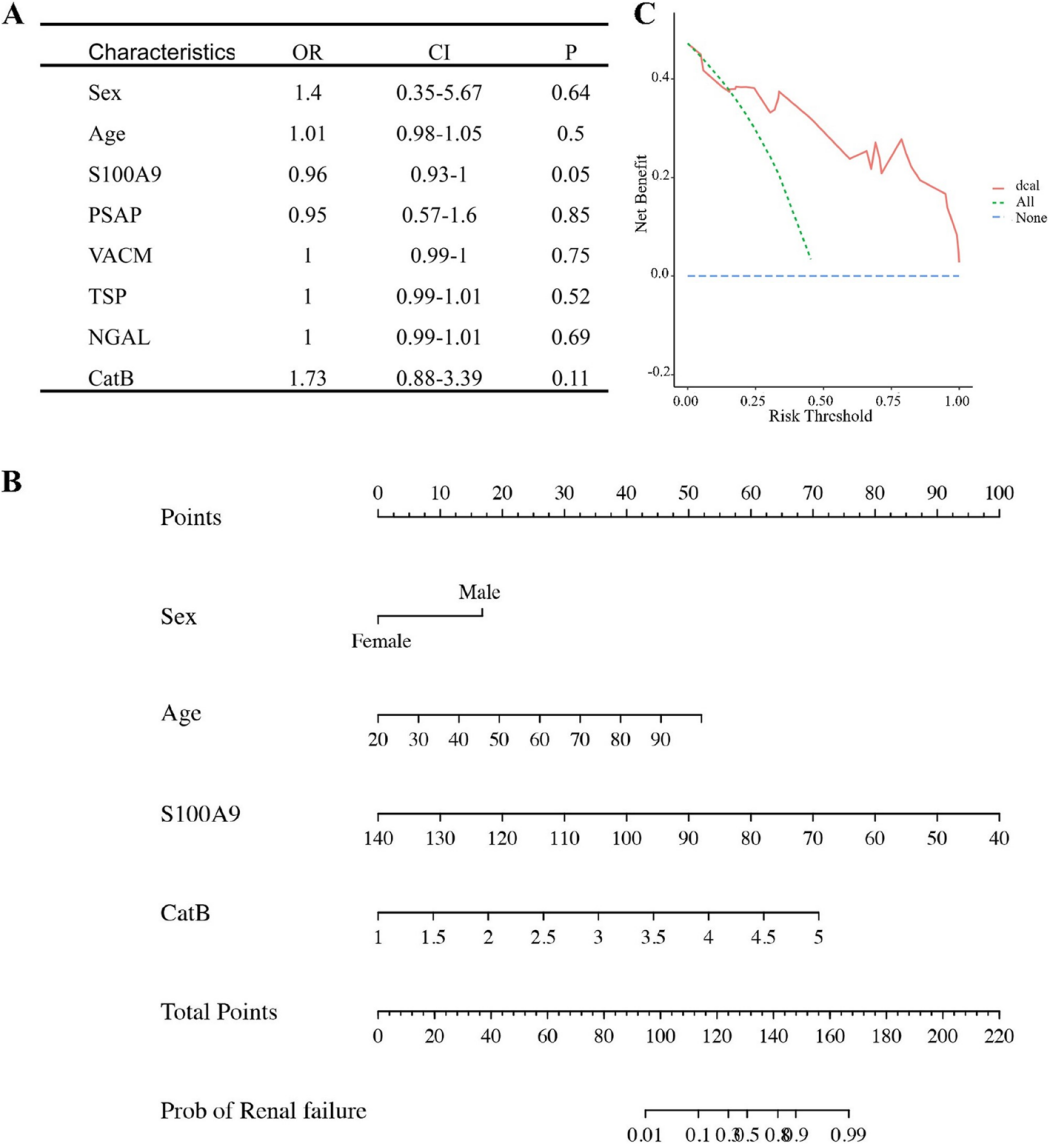
Investigation of the association between preoperative selected serum protein level and development of renal failure when patients presented to the hospital. **A** Summary of logistics regression model. **B** Nomogram for prediction of kidney failure during hospital presentation. **C** Plot of decision curve analysis of constructed model

Univariate logistic analyses were conducted to investigate possible correlations between biomarkers and single-organ dysfunction in septic patients before surgery. Figure 3A indicates a potential association between S100-A9 (*p* = 0.05) and CatB (*p* = 0.11) with preoperative renal failure in septic patients. Best subsets regression (BSR) based on the minimum Bayesian information criterions (BIC) and LASSO regression (LR) were further conducted to select the best variable combinations for the final prediction model for preoperative renal failure in septic patients (Supplemental Figure S2A, 2B). Sex, age, S100A9, and CatB were finally included to construct the prediction model. Supplemental Figure S2C shows the ROC curve and AUROC. The nomogram-histogram and decision curve analysis (DCA) showed that CatB and S100-A9 were predicted factors for renal failure in septic patients (Fig. 3B, C). The same process was used to predict the possibility of neurological dysfunction in septic patients before surgery (Supplemental Figure S3). Age (*p* = 0.14) and S100-A9 (*p* = 0.04) showed potential associations with neurological dysfunction in the univariate logistic analyses. After BSR and LASSO, age and S100-A9 were selected for the prediction model, which also showed a possible prediction effect of S100-A9. However, the DCA curve of this model indicated limited model benefits.

### Preoperative biomarkers predicted single organ dysfunction on D7

**Fig. 4:**
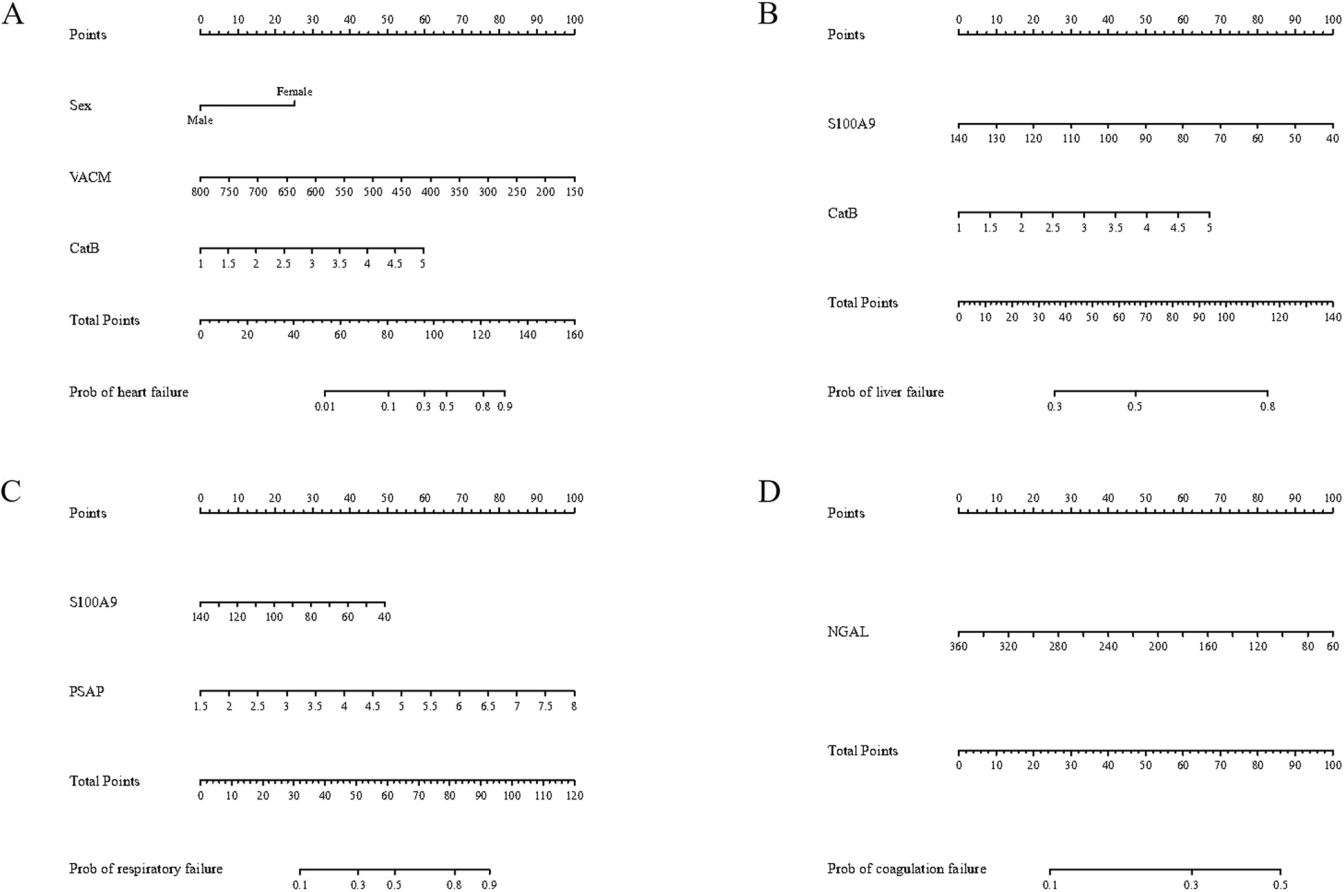
Prediction model of postoperative organ dysfunction. Following were nomogram and decision curve analyses that predict the probability of postoperative heart failure (**A**), liver failure (**B**), respiratory failure (**C**), and coagulation disorders (**D**)

Surgery can reverse sepsis-induced organ dysfunction. We examined whether specific biomarkers may predict the occurrence of postoperative organ dysfunction. Thus, we conducted further analyses to investigate potential correlations. Figure 4 and Supplemental Figure S4 show the nomogram-histogram and DCA curves for models predicting the occurrences of heart failure, liver dysfunction, respiratory failure, and coagulation dysfunction after surgery. CatB was a predictor factor in the prediction models of heart failure and liver dysfunction, whereas S100-A9 showed a predictive effect when predicting liver and respiratory failure. The nomogram-histogram also indicated an effect of prosaposin on respiratory failure, whereas VCAM-1 and NGAL were predicted factors for postoperative heart failure and coagulation dysfunction, respectively.

### Correlation between preoperative biomarkers and other outcomes

Analysis showed that these six biomarkers had little correlation with the length of ICU stay, length of hospital stay, SOFA, and APACHE II scores (Supplemental Figure S5). LR for the outcomes of septic patients showed that higher S100-A9 levels were associated with lower in-hospital mortality and 1-month mortality (Supplemental Figure S6).

### Apparent performance of the models in the cohort

We internally validated all organ dysfunction prediction models and found that the C-index for the preoperative diagnosis of renal failure model displayed good discrimination with a C-index of 0.898 (95% confidence interval: 0.843–0.954) and good calibration through bootstrapping validation (Supplemental Figure S7), while other models did not display good discrimination.

## Discussion

Sepsis is a series of clinical syndromes with high morbidity and mortality (Evans et al. 2021). Many sepsis-related biomarkers have been reported, but their diagnostic specificity and sensitivity remain unclear owing to the lack of consistent baseline characteristics (Simpson 2019; Kibe et al. 2011). In our study, patients’ conditions and biomarkers were continuously monitored from diagnosis to the postoperative period, and the results were self-controlled, which made up for the shortcomings of previous studies and increased the credibility of the data. Additionally, our study was the first to focus on the organ function statuses of patients with postoperative sepsis.

We found that serum CatB, S100-A9, VCAM-1, prosaposin, and NGAL can be used for preoperative diagnosis and postoperative prediction of organ dysfunction. Specifically, S100-A9 and CatB were indicators for preoperative diagnosis in septic patients.

We found S100-A9 to be a key marker in preoperative diagnosis and postoperative prediction of organ dysfunction, especially for renal failure. S100-A9 plays a key role in regulating inflammatory responses by stimulating leukocyte recruitment and inducing cytokine secretion (Santamaria-Kisiel et al. 2006). Studies have shown that S100-A9 was highly expressed in the serum of patients with pulmonary infectious diseases (Liu et al. 2021; Su et al. 2010). S100-A9 can be used as a candidate biomarker for diagnosis and follow-up as well as a predictor of treatment response to inflammatory diseases (Skopelja-Gardner et al. 2021; Ehrchen et al. 2009; Fontaine et al. 2011). To our knowledge, our study was the first to find that S100-A9 was a predictor in diagnosing sepsis-induced renal insufficiency. The mechanism may be related to renal tissue damage and inflammatory infiltration. S100-A9 was a valuable marker in predicting the occurrence of postoperative liver and respiratory failure, and higher S100-A9 values indicated a reduced possibility of liver and respiratory failure. Besides, S100-A9 was also found to predict hospital mortality and 1-month mortality in our study.

The effect of CatB was opposite that of S100-A9 in predicting preoperative diagnosis and postoperative prognosis in septic patients. CatB is a proteolytic enzyme involved in many diseases, such as acute and chronic inflammatory diseases (Mijanović et al. 2019; Man and Kanneganti 2016). In pathological conditions, cell damage caused by infection leads to CatB releasing into the cytoplasm, mediating cell necrosis and apoptosis (Nagakannan et al. 2021; Qi et al. 2016). Canbay et al. found that CatB was involved in inflammation and apoptosis caused by liver cholestasis after bile duct ligation, which ultimately led to liver fibrosis (Canbay et al. 2003). However, relevant studies on clinical samples are lacking. Our results showed that CatB was a possible predictor factor for preoperative diagnosis of renal failure in septic patients. And higher CatB levels indicated that patients were more likely to develop liver and heart failure. This was consistent with previous findings (Feng et al. 2018; Guo et al. 2020). However, further prospective controlled trial with larger samples is needed to confirm the causal correlation in the future.

Moreover, VCAM-1 may be used to postoperatively predict cardiac dysfunction. VCAM-1 has been implicated in neutrophil-mediated lung and liver injury (Raeburn et al. 2002; Figueras-Aloy et al. 2007; Li et al. 2020). We found that higher serum VCAM-1 levels indicated a lower possibility of heart failure, which contradicted the findings of previous studies. We suspect this may have been because septic patients are immunosuppressed. However, trials with larger samples are needed.

We found that NGAL was a marker for predicting postoperative coagulation dysfunction. NGAL has been reported to protect against bacterial infection and modulate oxidative stress and is a possible marker in acute kidney injury and cancer (Crescenzi et al. 2021; Shang and Wang 2017). However, no association was found between NGAL and renal failure in our study. Prosaposin acts as a regulator of lysosomal enzyme function and a secretory factor with neuroprotective and glial protective effects in the cell (Nabeka 2021). The ability of secreted prostaglandins to promote protective effects in the nervous system is known. However, we found the predicted effect of prosaposin on respiratory failure.

## Limitations

Our study had several limitations. First, it was a prospective study exploring novel biomarkers for the diagnosis or prognosis of organ dysfunction in surgery-related sepsis. Most patients enrolled in our study had biliary tract infections. The cohort was not representative of all septic patients; thus, the value of the biomarkers may not be easily generalizable. Second, risk factor analysis did not include all potential factors that affected medication adherence. Some possible aspects of nonadherence were not thoroughly informed such as the social support and other conditions. Third, it was a small pilot study. Although the robustness of our nomogram was examined extensively with internal validation using bootstrap testing, the generalizability was uncertain for other populations. In the future, the diagnostic and predictive performance of the explored biomarkers will be compared with the conventional markers of organ dysfunction (e.g., acute kidney injury markers). Finally, we did not continuously compare the varying tendencies of these biomarkers on D0 and D7, as well as the relationship with organ dysfunction. a longer follow-up process and continuous measurement of samples will be performed in the future. Therefore, further clinical studies with larger samples or animal research should be conducted to increase the credibility of the conclusions and rigor of the study.

## Conclusion

CatB, S100-A9, VCAM-1, prosaposin, and NGAL could be used for preoperative diagnosis and postoperative prediction of organ dysfunction. S100-A9 and Cat B were potential predict factors for preoperative prediction of renal failure. Monitoring both indicators may improve the accuracy of the diagnosis prediction of septic renal dysfunction. VCAM-1 may be used to postoperatively predict cardiac dysfunction. NGAL was a potential marker for predicting postoperative coagulation dysfunction. Besides, S100-A9 was also found to predict hospital mortality and 1-month mortality in our study.

### Supplementary Information

Supplementary Material 1: Supplemental Figure S1. Mass spectrometry analysis of the differentially expressed serum proteins in control and sepsis patients. (A) volcano diagram of patients with sepsis in D0 compared to control patients, showing some protein levels significantly decreased while some significantly increased; (B) volcano diagram of patients with sepsis in D7 compared to D0, showing some protein levels decreased while some increased. D0: blood samples of patients with sepsis at admission; D7: blood samples of patients with sepsis on the seventh day after surgery. Supplemental Figure S2. Variables selection procedure for logistics regression model predicting probability of renal failure when patients presented to the hospital. A. Procedure of best subset selection. B. Process of lasso regression. C. Receiver operating characteristic (ROC) curve of constructed model. Supplemental Figure S3. Investigation of the association between preoperative selected serum protein level and development of central nervous system (CNS) dysfunction when patients presented to the hospital. A. Summary of logistics regression model. B. Procedure of best subset selection. C. Process of lasso regression. D. ROC curve of constructed model. E. Nomogram for prediction of CNS dysfunction during hospital presentation. F. Plot of decision curve analysis of constructed model. Supplemental Figure S4. DCA curves that predicting the probability of postoperative heart failure (A), liver failure (B), respiratory failure (C), and coagulation disorders (D). Supplemental Figure S5. The association between preoperative selected serum protein level and other clinical outcomes. A. The correlogram of selected preoperative serum protein level and clinical outcomes of sepsis patients, including length of ICU stay, hospital stay, SOFA score, and APACHEII score. More intense colors indicated more extreme correlations. B&C. Forest plot indicating the effect of gender, age, and selected serum protein level on sepsis patients’ clinical outcome at discharge (B) and 30 days after discharge (C). Supplemental Figure S6. Variable selection procedure for the logistic regression model predicting probability of death within 30 days after discharge. A. Best subset selection procedure. B. LASSO regression process. C. ROC curve of the constructed model. Supplemental Figure S7. Calibration curve of preoperative diagnosis of renal failure model.

## Data Availability

No datasets were generated or analysed during the current study.

## References

[CR1] Canbay A, Guicciardi ME, Higuchi H, Feldstein A, Bronk SF, Rydzewski R, et al. Cathepsin B inactivation attenuates hepatic injury and fibrosis during cholestasis. J Clin Investig. 2003;112(2):152–9. 10.1172/JCI17740. 12865404 10.1172/JCI17740 PMC164289

[CR2] Chiu C, Legrand M. Epidemiology of sepsis and septic shock. Curr Opin Anesthesiol. 2021;34(2):71–6. 10.1097/ACO.0000000000000958. 10.1097/ACO.0000000000000958 33492864

[CR3] Crescenzi E, Leonardi A, Pacifico F. NGAL as a potential target in tumor microenvironment. Int J Mol Sci. 2021;22(22):12333. 10.3390/ijms222212333. 34830212 10.3390/ijms222212333 PMC8623964

[CR4] Ehrchen JM, Sunderkötter C, Foell D, Vogl T, Roth J. The endogenous Toll-like receptor 4 agonist S100A8/S100A9 (calprotectin) as innate amplifier of infection, autoimmunity, and cancer. J Leukoc Biol. 2009;86(3):557–66. 10.1189/jlb.1008647. 19451397 10.1189/jlb.1008647

[CR5] Evans L, Rhodes A, Alhazzani W, Antonelli M, Coopersmith CM, French C, et al. Surviving sepsis campaign: international guidelines for management of sepsis and septic shock 2021. Intensive Care Med. 2021;47(11):1181–247. 10.1007/s00134-021-06506-y. 34599691 10.1007/s00134-021-06506-y PMC8486643

[CR6] Feng P, Zhu W, Chen N, Li P, He K, Gong J. Cathepsin B in hepatic Kupffer cells regulates activation of TLR4-independent inflammatory pathways in mice with lipopolysaccharide-induced sepsis. Nan Fang Yi Ke Da Xue Xue Bao. 2018;38(12):1465–71. 10.12122/j.issn.1673-4254.2018.12.11. 30613015 10.12122/j.issn.1673-4254.2018.12.11 PMC6744205

[CR7] Figueras-Aloy J, Gómez-López L, Rodríguez-Miguélez JM, Salvia-Roiges MD, Jordán-García I, Ferrer-Codina I, et al. Serum soluble ICAM-1, VCAM-1, L-selectin, and P-selectin levels as markers of infection and their relation to clinical severity in neonatal sepsis. Am J Perinatol. 2007;24(6):331–8. 10.1055/s-2007-981851. 17564956 10.1055/s-2007-981851

[CR8] Fleischmann C, Scherag A, Adhikari NK, Hartog CS, Tsaganos T, Schlattmann P, et al. Assessment of global incidence and mortality of hospital-treated sepsis. Current estimates and limitations. Am J Respir Criti Care Med. 2016;193(3):259–72. 10.1164/rccm.201504-0781OC. 10.1164/rccm.201504-0781OC 26414292

[CR9] Fontaine M, Pachot A, Larue A, Mougin B, Landelle C, Venet F, et al. Delayed increase of S100A9 messenger RNA predicts hospital-acquired infection after septic shock. Crit Care Med. 2011;39(12):2684–90. 10.1097/CCM.0b013e3182282a40. 21765347 10.1097/CCM.0b013e3182282a40

[CR10] González Del Castillo J, Clemente C, Candel FJ, Martín-Sánchez FJ. New sepsis criteria: do they replace or complement what is known in the approach to the infectious patient? Rev Esp Quimioter. 2017;30(Suppl 1):48–51. 28882016

[CR11] Guo R, Yu Q, Liong EC, Fung ML, Tipoe GL. Cathepsin-B dependent autophagy ameliorates steatoheaptitis in chronic exercise rats. Histol Histopathol. 2020;35(8):833–47. 10.14670/HH-18-204. 31975365 10.14670/HH-18-204

[CR12] Hotchkiss RS, Monneret G, Payen D. Sepsis-induced immunosuppression: from cellular dysfunctions to immunotherapy. Nat Rev Immunol. 2013;13(12):862–74. 10.1038/nri3552. 24232462 10.1038/nri3552 PMC4077177

[CR13] Jiao J, Gao M, Zhang H, Wang N, Xiao Z, Liu K, et al. Identification of potential biomarkers by serum proteomics analysis in rats with sepsis. Shock. 2014;42(1):75–81. 10.1097/SHK.0000000000000173. 24667622 10.1097/SHK.0000000000000173

[CR14] Kibe S, Adams K, Barlow G. Diagnostic and prognostic biomarkers of sepsis in critical care. J Antimicrob Chemother. 2011;66 Suppl:ii33–40. 10.1093/jac/dkq523. 21398306 10.1093/jac/dkq523

[CR15] Konradsen S, Lien AH. New sepsis criteria may lead to delayed treatment. Tidsskr Nor Laegeforen. 2017;137(9):609–10. 10.4045/tidsskr.17.0114. 28468474 10.4045/tidsskr.17.0114

[CR16] Li Y, Huang X, Guo F, Lei T, Li S, Monaghan-Nichols P, et al. TRIM65 E3 ligase targets VCAM-1 degradation to limit LPS-induced lung inflammation. J Mol Cell Biol. 2020;12(3):190–201. 10.1093/jmcb/mjz077. 31310649 10.1093/jmcb/mjz077 PMC7181722

[CR17] Liu Q, Li R, Li Q, Luo B, Lin J, Lyu L. High levels of plasma S100A9 at admission indicate an increased risk of death in severe tuberculosis patients. J Clin Tuberc Other Mycobact Dis. 2021;25:100270. 10.1016/j.jctube.2021.100270. 34849408 10.1016/j.jctube.2021.100270 PMC8609153

[CR18] Man SM, Kanneganti TD. Regulation of lysosomal dynamics and autophagy by CTSB/cathepsin B. Autophagy. 2016;12(12):2504–5. 10.1080/15548627.2016.1239679. 27786577 10.1080/15548627.2016.1239679 PMC5173259

[CR19] Marshall JC, Cook DJ, Christou NV, Bernard GR, Sprung CL, Sibbald WJ. Multiple organ dysfunction score: a reliable descriptor of a complex clinical outcome. Crit Care Med. 1995;23(10):1638–52. 10.1097/00003246-199510000-00007. 7587228 10.1097/00003246-199510000-00007

[CR20] Mijanović O, Branković A, Panin AN, Savchuk S, Timashev P, Ulasov I, et al. Cathepsin B: a sellsword of cancer progression. Cancer Lett. 2019;449:207–14. 10.1016/j.canlet.2019.02.035. 30796968 10.1016/j.canlet.2019.02.035 PMC6488514

[CR21] Nabeka H. Prosaposin, a neurotrophic factor, protects neurons against kainic acid-induced neurotoxicity. Anat Sci Int. 2021;96(3):359–69. 10.1007/s12565-021-00605-y. 33534127 10.1007/s12565-021-00605-y

[CR22] Nagakannan P, Islam MI, Conrad M, Eftekharpour E. Cathepsin B is an executioner of ferroptosis. BBA-Mol Cell Res. 2021;1868(3):118928. 10.1016/j.bbamcr.2020.118928. 10.1016/j.bbamcr.2020.118928 33340545

[CR23] Opal SM, Wittebole X. Biomarkers of infection and sepsis. Crit Care Clin. 2020;36(1):11–22. 10.1016/j.ccc.2019.08.002. 31733673 10.1016/j.ccc.2019.08.002

[CR24] Philippon AL, Freund Y. Research on sepsis biomarkers in the emergency department: what now, what next? Emergencias. 2019;31(5):302–3. 31625300

[CR25] Pierrakos C, Vincent JL. Sepsis biomarkers: a review. Crit Care. 2010;14(1):R15. 10.1186/cc8872. 20144219 10.1186/cc8872 PMC2875530

[CR26] Qi X, Man SM, Malireddi RK, Karki R, Lupfer C, Gurung P, et al. Cathepsin B modulates lysosomal biogenesis and host defense against Francisella novicida infection. J Exp Med. 2016;213(10):2081–97. 10.1084/jem.20151938. 27551156 10.1084/jem.20151938 PMC5030800

[CR27] Raeburn CD, Calkins CM, Zimmerman MA, Song Y, Ao L, Banerjee A, et al. ICAM-1 and VCAM-1 mediate endotoxemic myocardial dysfunction independent of neutrophil accumulation. Am J Physiol Regul Integr Comp Physiol. 2002;283(2):R477-486. 10.1152/ajpregu.00034.2002. 12121861 10.1152/ajpregu.00034.2002

[CR28] Santamaria-Kisiel L, Rintala-Dempsey AC, Shaw GS. Calcium-dependent and -independent interactions of the S100 protein family. Biochem J. 2006;396(2):201–14. 10.1042/BJ20060195. 16683912 10.1042/BJ20060195 PMC1462724

[CR29] Shang W, Wang Z. The update of NGAL in acute kidney injury. Curr Protein Pept Sci. 2017;18(12):1211–7. 10.2174/1389203717666160909125004. 27634444 10.2174/1389203717666160909125004

[CR30] Simpson SQ. New sepsis criteria: a change we should not make. Chest. 2016;149(5):1117–8. 10.1016/j.chest.2016.02.653. 26927525 10.1016/j.chest.2016.02.653

[CR31] Simpson SQ. Sepsis biomarkers and physician judgment in the emergency room. Crit Care Med. 2019;47(11):1656–7. 10.1097/CCM.0000000000003983. 31609261 10.1097/CCM.0000000000003983

[CR32] Singer M, Deutschman CS, Seymour CW, Shankar-Hari M, Annane D, Bauer M, et al. The third international consensus definitions for sepsis and septic shock (Sepsis-3). JAMA. 2016;315(8):801–10. 10.1001/jama.2016.0287. 26903338 10.1001/jama.2016.0287 PMC4968574

[CR33] Skopelja-Gardner S, Tai J, Sun X, Tanaka L, Kuchenbecker JA, Snyder JM, et al. Acute skin exposure to ultraviolet light triggers neutrophil-mediated kidney inflammation. Proc Natl Acad Sci USA. 2021;118(3):e2019097118. 10.1073/pnas.2019097118. 33397815 10.1073/pnas.2019097118 PMC7826360

[CR34] Su YJ, Xu F, Yu JP, Yue DS, Ren XB, Wang CL. Up-regulation of the expression of S100A8 and S100A9 in lung adenocarcinoma and its correlation with inflammation and other clinical features. Chin Med J. 2010;123(16):2215–20. 20819668

