# Supplementary material for: Exploring biomarkers for diagnosing and predicting organ dysfunction in patients with perioperative sepsis: a preliminary investigation
Source: Perioper Med (Lond). 2024 Jul 24;13:81. doi: 10.1186/s13741-024-00438-z (PMC11267738; doi:10.1186/s13741-024-00438-z)
Supplement: Supplementary file 1 — Supplementary Material 1: Supplemental Figure S1. Mass spectrometry analysis of the differentially expressed serum proteins in control and sepsis patients. (A) volcano diagram of patients with sepsis in D0 compared to control patients, showing some protein levels significantly decreased while some significantly increased; (B) volcano diagram of patients with sepsis in D7 compared to D0, showing some protein levels decreased while some increased. D0: blood samples of patients with sepsis at admission; D7: blood samples of patients with sepsis on the seventh day after surgery. Supplemental Figure S2. Variables selection procedure for logistics regression model predicting probability of renal failure when patients presented to the hospital. A. Procedure of best subset selection. B. Process of lasso regression. C. Receiver operating characteristic (ROC) curve of constructed model. Supplemental Figure S3. Investigation of the association between preoperative selected serum protein level and development of central nervous system (CNS) dysfunction when patients presented to the hospital. A. Summary of logistics regression model. B. Procedure of best subset selection. C. Process of lasso regression. D. ROC curve of constructed model. E. Nomogram for prediction of CNS dysfunction during hospital presentation. F. Plot of decision curve analysis of constructed model. Supplemental Figure S4. DCA curves that predicting the probability of postoperative heart failure (A), liver failure (B), respiratory failure (C), and coagulation disorders (D). Supplemental Figure S5. The association between preoperative selected serum protein level and other clinical outcomes. A. The correlogram of selected preoperative serum protein level and clinical outcomes of sepsis patients, including length of ICU stay, hospital stay, SOFA score, and APACHEII score. More intense colors indicated more extreme correlations. B&C. Forest plot indicating the effect of gender, age, and selected serum protein le [file 13741_2024_438_MOESM1_ESM.pdf]

Contents

Supplemental Figure 1……………………………………………2

Supplemental Figure 2……………………………………………3

Supplemental Figure 3……………………………………………4

Supplemental Figure 4…………………………………………….5

Supplemental Figure 5……………………………………………..6

Supplemental Figure 6……………………………………………..7

Supplemental Figure 7……………………………………………..8

Supplemental Figure 1

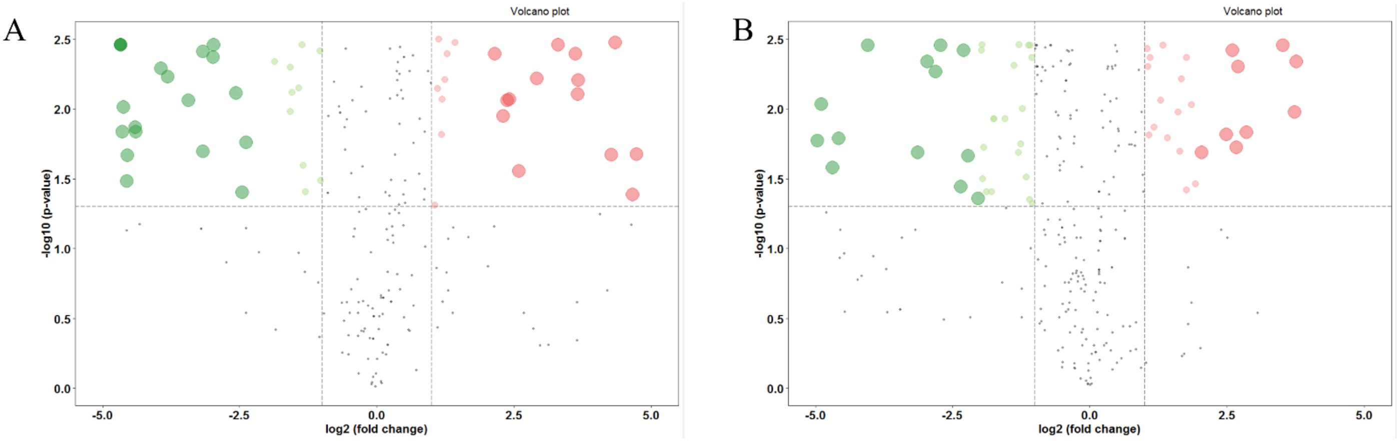

Mass spectrometry analysis of the differentially expressed serum proteins in control and sepsis patients. (A) volcano diagram of patients with sepsis in D0 compared to control patients, showing some protein levels significantly decreased while some significantly increased; (B) volcano diagram of patients with sepsis in D7 compared to D0, showing some protein levels decreased while some increased. D0: blood samples of patients with sepsis at admission; D7: blood samples of patients with sepsis on the seventh day after surgery.

Supplemental Figure 2

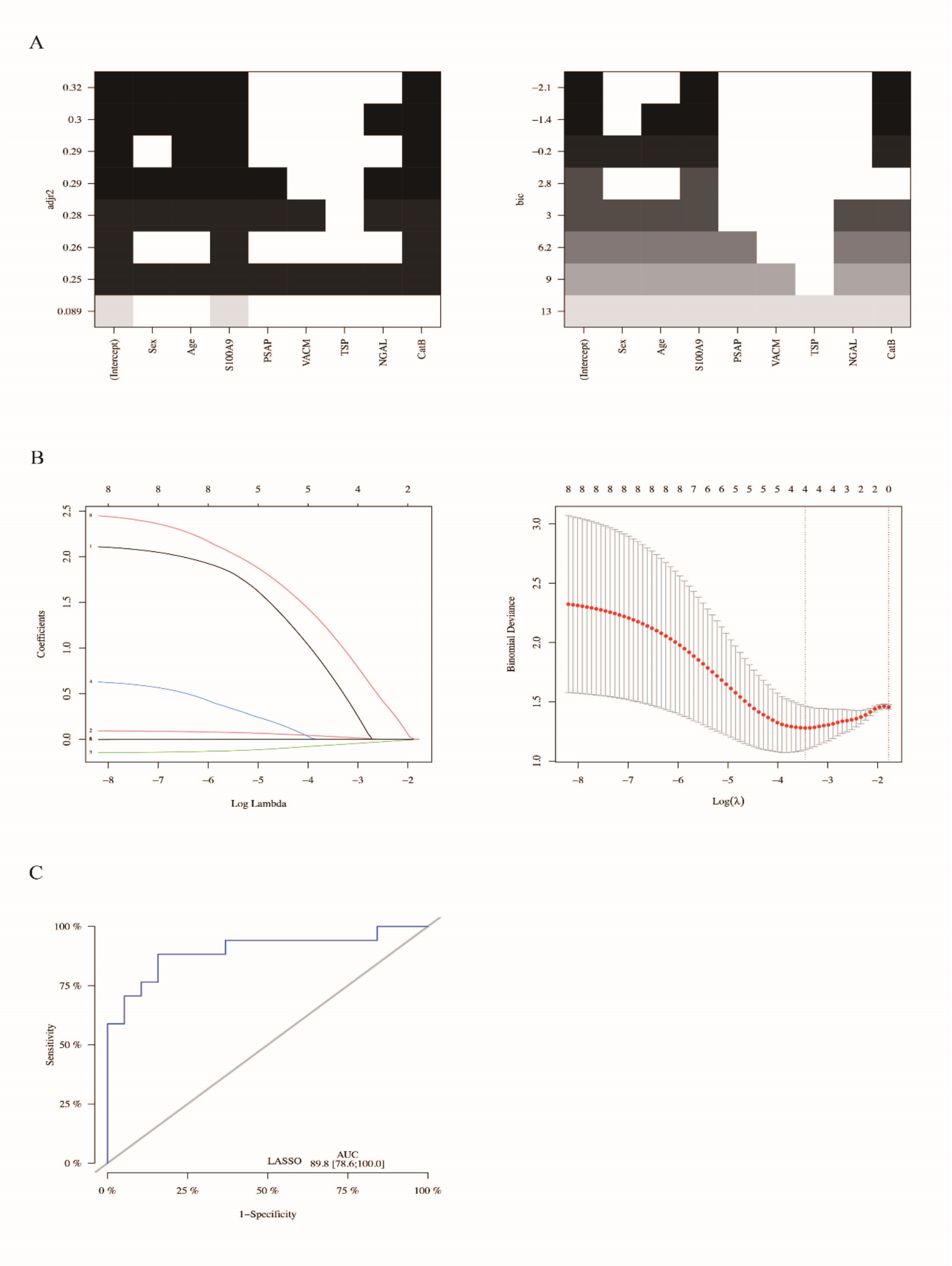

Variables selection procedure for logistics regression model predicting probability of renal failure when patients presented to the hospital. **A.** Procedure of best subset selection. **B.** Process of lasso regression. **C.** Receiver operating characteristic (ROC) curve of constructed model.

Supplemental Figure 3

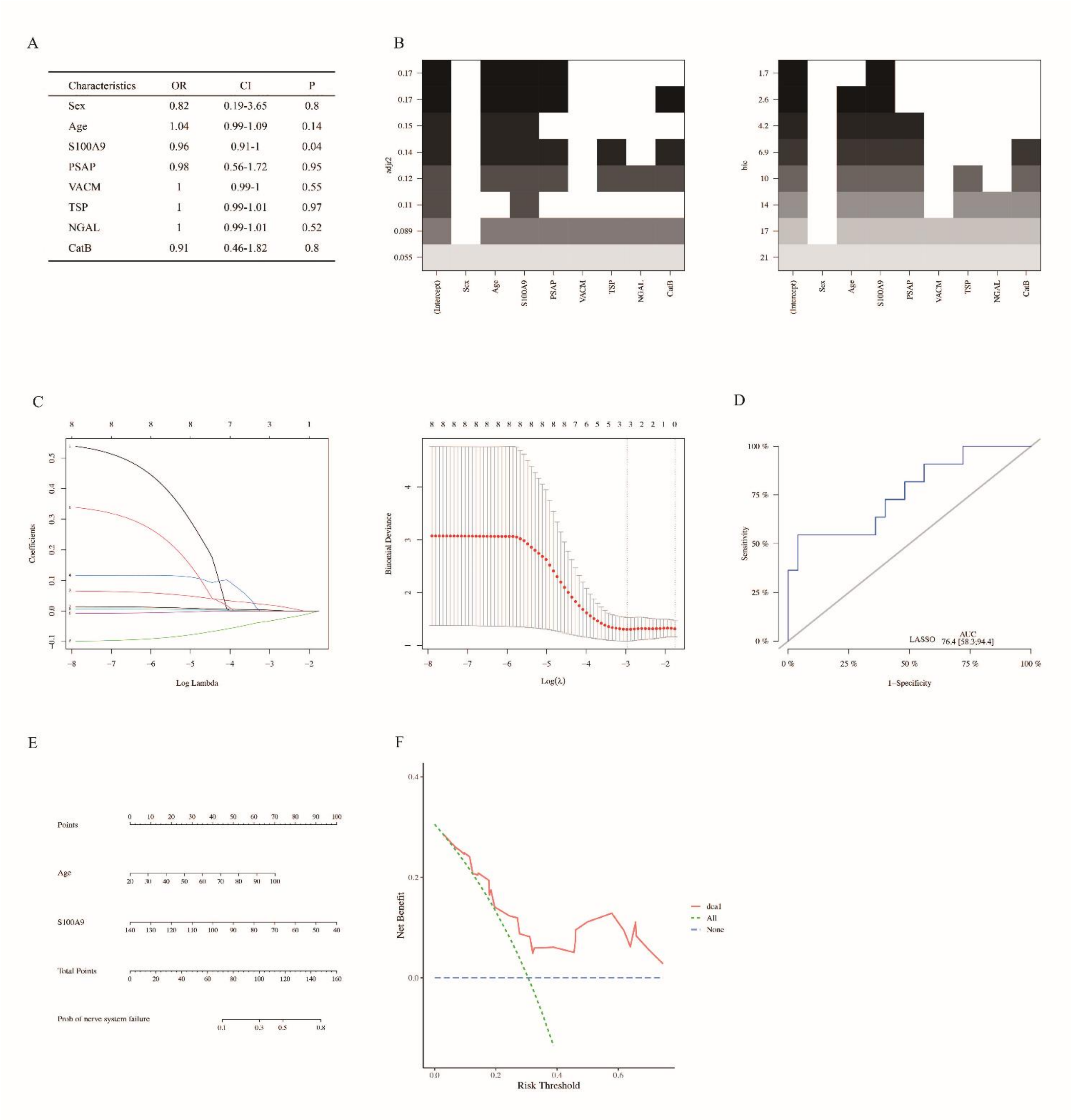

Characteristics	OR	CI	P
Sex	0.82	0.19-3.65	0.8
Age	1.04	0.99-1.09	0.14
S100A9	0.96	0.91-1	0.04
PSAP	0.98	0.56-1.72	0.95
VACM	1	0.99-1	0.55
TSP	1	0.99-1.01	0.97
NGAL	1	0.99-1.01	0.52
CatB	0.91	0.46-1.82	0.8

Investigation of the association between preoperative selected serum protein level and development of central nervous system (CNS) dysfunction when patients presented to the hospital. **A.** Summary of logistics regression model. **B.** Procedure of best subset selection. **C.** Process of lasso regression. **D.** ROC curve of constructed model. **E.** Nomogram for prediction of CNS dysfunction during hospital presentation. **F**. Plot of decision curve analysis of constructed model.

Supplemental Figure 4

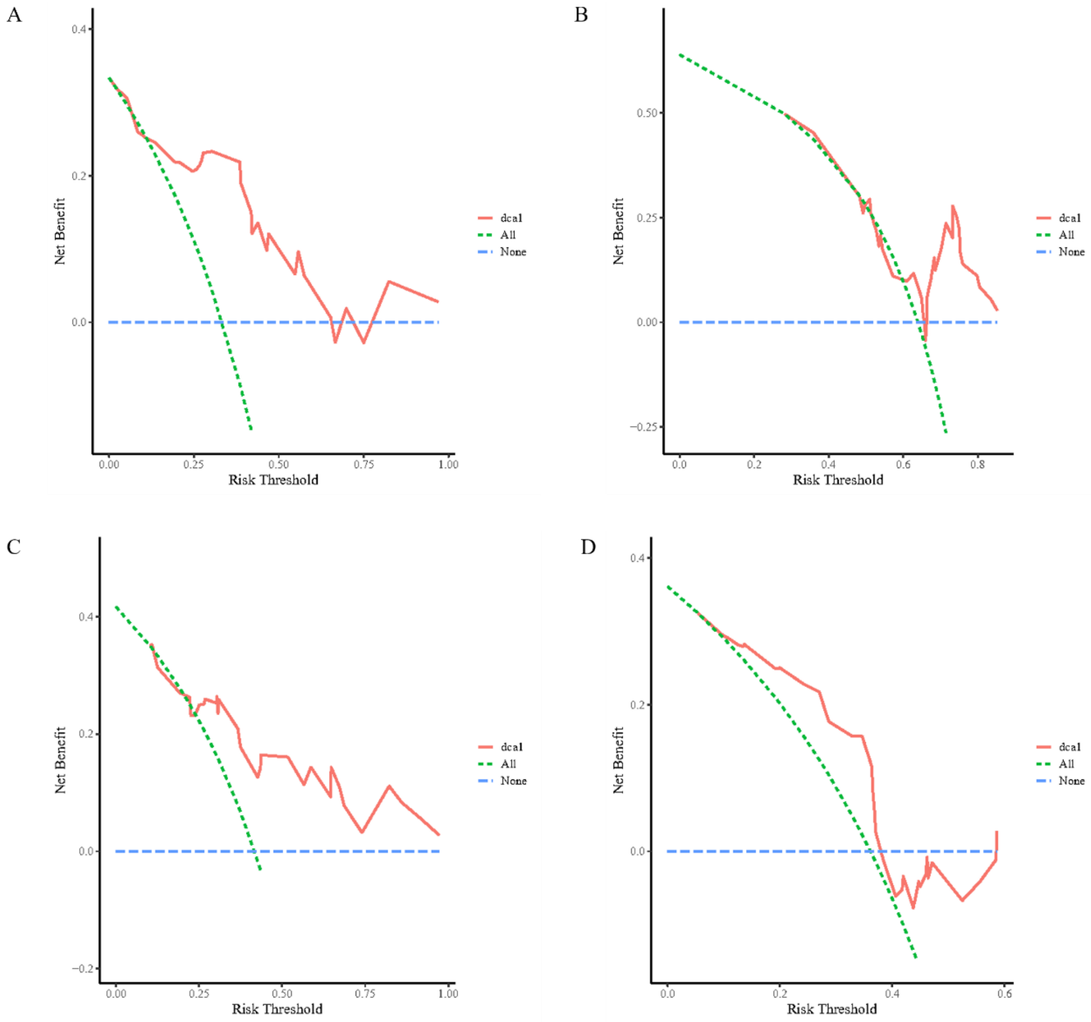

DCA curves that predicting the probability of postoperative heart failure (A), liver failure (B), respiratory failure (C), and coagulation disorders (D).

Supplemental Figure 5

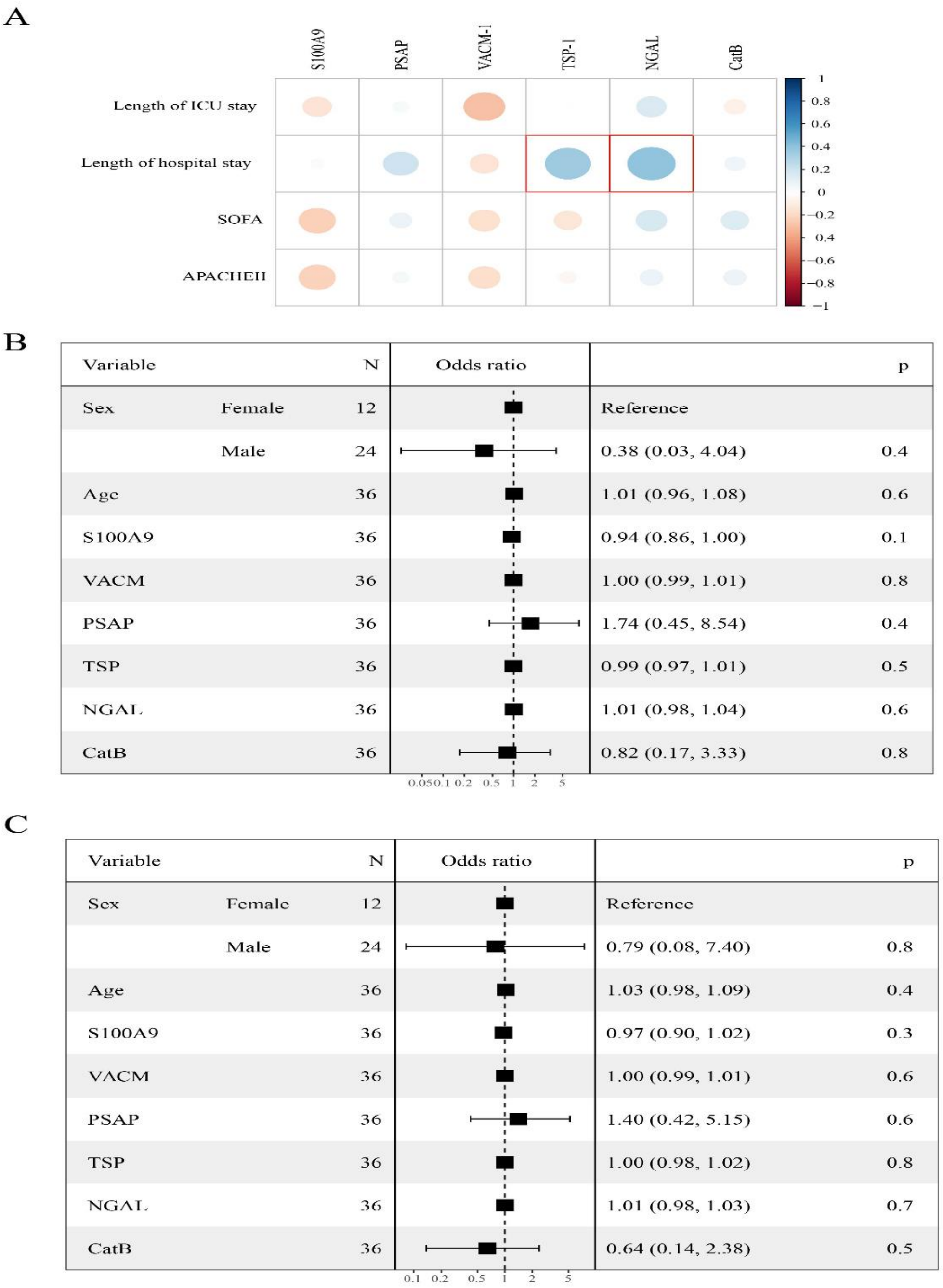

The association between preoperative selected serum protein level and other clinical outcomes. **A.** The correlogram of selected preoperative serum protein level and clinical outcomes of sepsis patients, including length of ICU stay, hospital stay, SOFA score, and APACHEII score. More intense colors indicated more extreme correlations. B&C. Forest plot indicating the effect of gender, age, and selected serum protein level on sepsis patients' clinical outcome at discharge (**B**) and 30 days after discharge (**C**).

Supplemental Figure 6

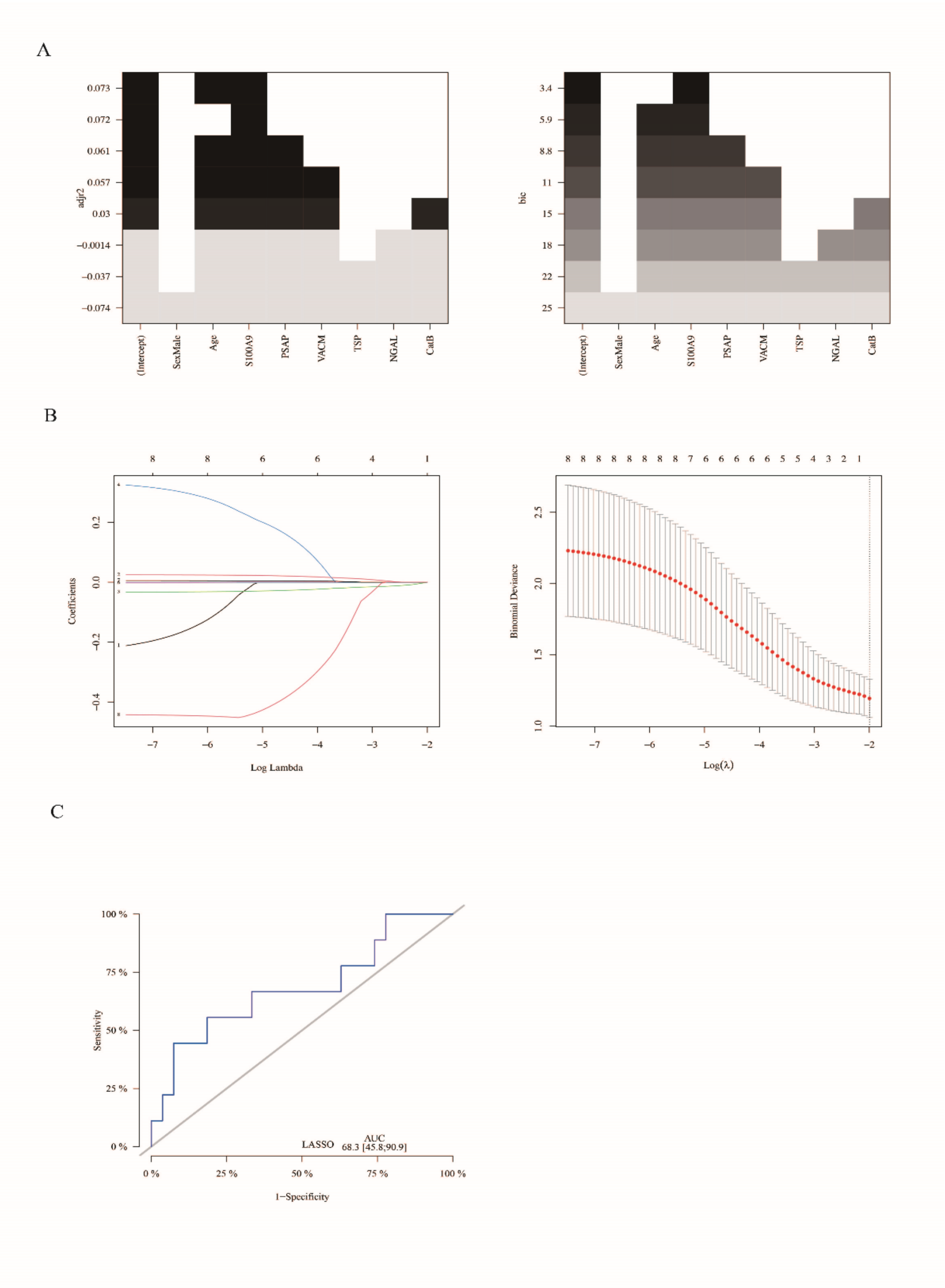

Variable selection procedure for the logistic regression model predicting probability of death within 30 days after discharge. **A.** Best subset selection procedure. **B.** LASSO regression process. **C.** ROC curve of the constructed model.

Supplemental Figure 7

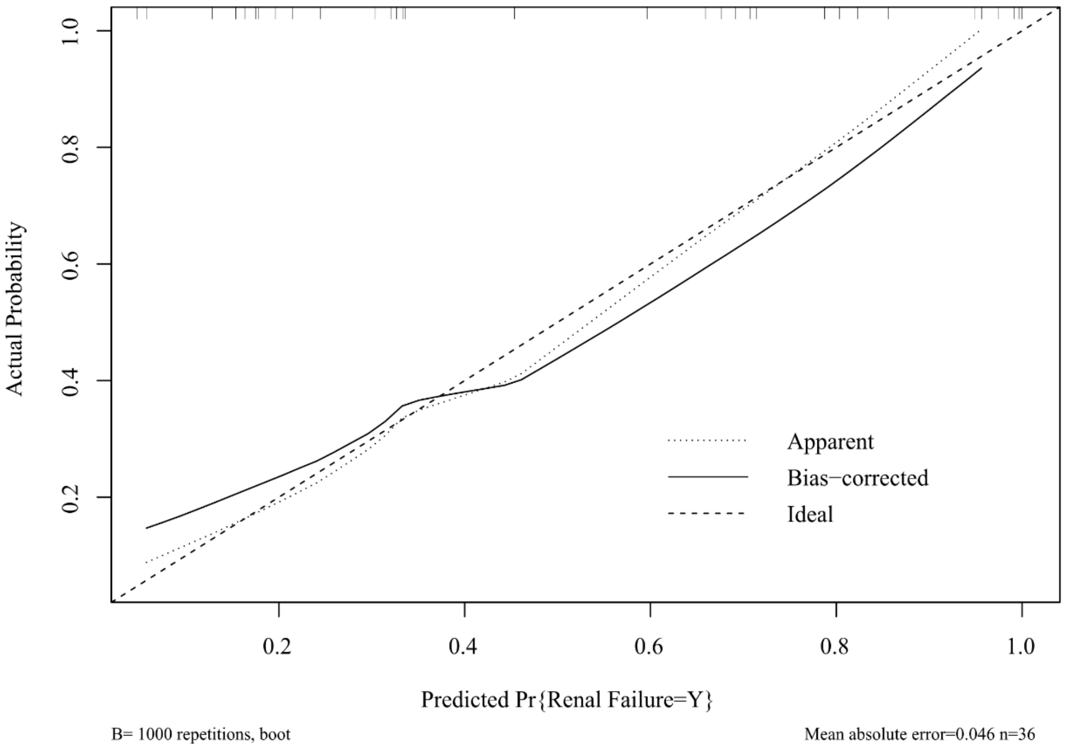

Calibration curve of preoperative diagnosis of renal failure model
